# A major QTL controls susceptibility to spinal curvature in the *curveback *guppy

**DOI:** 10.1186/1471-2156-12-16

**Published:** 2011-01-26

**Authors:** Kristen F Gorman, Julian K Christians, Jennifer Parent, Roozbeh Ahmadi, Detlef Weigel, Christine Dreyer, Felix Breden

**Affiliations:** 1 Department of Biological Sciences, Simon Fraser University, Burnaby, British Columbia, Canada; 2 Department of Molecular Biology at the Max Planck Institute for Developmental Biology, Tübingen, Germany

## Abstract

**Background:**

Understanding the genetic basis of heritable spinal curvature would benefit medicine and aquaculture. Heritable spinal curvature among otherwise healthy children (*i.e. *Idiopathic Scoliosis and Scheuermann kyphosis) accounts for more than 80% of all spinal curvatures and imposes a substantial healthcare cost through bracing, hospitalizations, surgery, and chronic back pain. In aquaculture, the prevalence of heritable spinal curvature can reach as high as 80% of a stock, and thus imposes a substantial cost through production losses. The genetic basis of heritable spinal curvature is unknown and so the objective of this work is to identify quantitative trait loci (QTL) affecting heritable spinal curvature in the *curveback *guppy. Prior work with *curveback *has demonstrated phenotypic parallels to human idiopathic-type scoliosis, suggesting shared biological pathways for the deformity.

**Results:**

A major effect QTL that acts in a recessive manner and accounts for curve susceptibility was detected in an initial mapping cross on LG 14. In a second cross, we confirmed this susceptibility locus and fine mapped it to a 5 cM region that explains 82.6% of the total phenotypic variance.

**Conclusions:**

We identify a major QTL that controls susceptibility to curvature. This locus contains over 100 genes, including MTNR1B, a candidate gene for human idiopathic scoliosis. The identification of genes associated with heritable spinal curvature in the *curveback *guppy has the potential to elucidate the biological basis of spinal curvature among humans and economically important teleosts.

## Background

Idiopathic-type spinal curvature is a heritable condition that occurs during development without other structural malformations that would be indicative of a congenital defect. The deformity is observed among humans and teleosts, but not among quadrupedal animals [1]. The phenotype is a consequence of genetic, biomechanical, and environmental factors that affect the spine during development.

### Heritable idiopathic-type spinal curvature among humans

Idiopathic-type spinal curvature is the primary defect in the Idiopathic Scoliosis syndrome (IS) and Scheuermann kyphosis, and is also associated with other heritable disorders such as Prader-Willi syndrome and Turner syndrome. The extent to which etiological factors for curvature are shared between these developmental syndromes is unknown. Scheuermann kyphosis and IS may share genetic factors, based on familial clustering of the two pathologies [2], and/or common pathological processes [3,4]. However, the syndromes differ morphologically; IS occurs in all three planes of the body and Scheuermann kyphosis is primarily a sagittal defect.

The global prevalence of IS among children is 0.5-10% (reviewed in [5]), and the prevalence for Scheuermann kyphosis is 4-10% [6]. Neither the genetic basis nor the biological processes involved in the aetiology of either of these idiopathic-type spinal curvatures are known. The poor understanding of causative factors is due to trait complexity, including a high degree of phenotypic variability coinciding with growth, and a historic lack of a genetic and developmental animal model.

Recent work with the guppy (*Poecilia reticulata*) *curveback *lineage has demonstrated morphological and developmental similarities to the human IS syndrome and Scheuermann kyphosis [7-10]. Considering that teleosts share most developmental pathways, physiological mechanisms and organ systems with humans, it is likely that there are shared biological pathways for spinal curvature.

### Heritable spinal curvature among teleosts

Spinal curvature is the most common morphological deformity among teleosts and can be caused by a variety of influences including nutritional, environmental, and genetic factors (reviewed in [10]). Heritable spinal curvature has been noted among laboratory (*e.g. *swordtail, guppy, medaka) and aquaculture (*e.g. *seabream, salmon, trout, sea bass) stocks. Among aquaculture stocks, spinal curvature can detract from the value of fish, and has prevented aquaculture from achieving optimal production [11-16]. The incidence of spinal curvature among aquaculture stocks ranges from 1-81%, depending on the species [16,17]. Although environmental factors have been associated with spinal curvature, several lines of evidence indicate that a genetic component contributes to a significant portion of these deformities in teleosts [13,16,18-20]. However, no genes have as yet been identified that contribute to this deformity, even though such identification would enable aquaculture populations to be screened for alleles causing deformity, which could then be eliminated via marker assisted selection.

**Figure 1:**
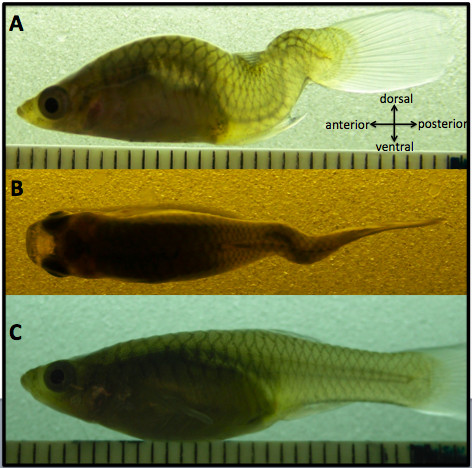
**The *curveback *phenotype**. The *curveback *phenotype is defined as a curve that occurs primarily in the sagittal plane of the fish as an anterior lordosis (ventrally directed curve) and posterior kyphosis (dorsally directed curve) (**A**). Some fish also exhibit deviation of the spine on the coronal plane (**B**). **C **shows a non-curved fish. All photos are of adult female fish. Scale is in mm.

The *curveback *guppy lineage has a phenotype that has been extensively characterized, consisting of a heritable, idiopathic-type curve that has a propensity to increase in magnitude with growth [7]. Guppy offspring are born with a fully ossified skeleton after ~3 weeks of gestation (guppies are live-bearers), and in the *curveback *lineage, curvature begins after birth and is generally stable by sexual maturity (approximately a month after birth). The *curveback *phenotype occurs primarily on the sagittal plane of the fish as an anterior lordosis and a posterior kyphosis, both of variable magnitude. In addition, some individuals exhibit coronal deviation of the spine **(Figure **1).

As described for human idiopathic-type scoliosis syndromes, there is extensive variability in the *curveback *population for curve magnitude, as well as for the propensity for a curve to progress, and for the rate of progression. Moreover, our initial survey of inheritance for curvature suggests that it is a complex trait under the control of multiple genes [7]. Therefore, we used quantitative trait locus (QTL) mapping to identify genomic regions that are associated with curvature. Genes that are associated with idiopathic-type scoliosis in *curveback *will suggest candidate genes and biological pathways that may be involved in the human and other teleost curve phenotypes. Here we describe the mapping of chromosomal regions associated with spinal curvature in the *curveback *guppy.

## Results

### Detection of QTL for idiopathic-type spinal curvature in *curveback*

QTL were detected using mapping progeny generated from Cross 1. From this cross 30 F1 offspring were produced, none of which were curved, indicating that curvature is a recessive trait. In the 129 backcross (BC) progeny, 48% had curves of variable magnitude whereas in the 286 F2 progeny, 22% showed curves of varying magnitude. The percent curved fish among BC and F2 progeny suggests that curvature is caused by a major recessive locus that is inherited in a Mendelian recessive manner. However, variation for curve magnitude among curved progeny suggests the presence of modifier alleles. The distribution of curve magnitude among BC and F2 offspring is given in **Table **1.

**Table 1:** The distribution of curve magnitude among *curveback *mapping offspring

	Qualitative value for curve magnitude									Total offspring	Total curved	Percent curved
	0	1		2		3		4				
Backcross	67	32	52%	23	37%	5	8%	2	3%	129	62	48%
F2 - Cross 1	224	40	64%	16	26%	6	10%	0	n/a	286	62	22%
F2 - Cross 2	200	11	19%	35	59%	10	17%	3	5%	259	59	29%

Backcross offspring are from Cross 1. Fish were scored for curve magnitude using a qualitative scale [7]: 0 = non-curved to 4 = extreme curve. Mapping offspring are euthanized at a minimum of 3 months past birth (sexual maturity is at about 1 month past birth), photographed, and frozen in buffered (EDTA) ethanol. The same person scored each individual alive, and then scores were confirmed in photos. Among each phenotypic class (score), the proportion of fish among curved individuals is shown. All fish were photographed on a light table with a digital camera under 3X magnification.

Of the 376 SNPs tested on BC offspring of Cross 1, 168 markers (44.6%) were polymorphic, 151 of which were assigned to 23 guppy linkage groups by Tripathi, *et al., *2009 [21] (Additional file 1). With the exception of LG 3, which has one marker, the number of markers per LG ranges from 3-14 and the size of intervals between markers ranges from 0.1-19.57 cM (**Table **2). Interval mapping identified one major effect QTL on LG14 that has a significant association with curvature (LOD 9.4; F = 57.66, chromosome-wide 1% significance threshold F = 9.03). The estimated location of this QTL is near Marker 0294 on LG14 (15.5 cM) [21]. The allele substitution effect estimated to be 1.39 (0.17 SE). At Marker 0294 the alleles are fixed in the parents and 94% of the curved individuals and 25% of non-curved individuals were homozygous for the allele of the curved parent, suggesting that the QTL contains a gene or genes for curve predisposition. Linkage group 3 was omitted from the interval analysis because it only contained one marker, which is insufficient for interval mapping. One-way ANOVAs showed no association between the marker on LG3 and curve magnitude [F(1,82) = 1.31, p = 0.26], or between the markers that could not be assigned to a linkage group and curve magnitude (results not shown). We did not detect any other QTL with the single or two QTL model.

**Table 2:** Marker coverage for each linkage group in interval analysis

Linkage group	Number of markers	Smallest interval (cM)	Largest interval (cM)	Total length (cM)
**1**	4	7.5	15.92	36.04
**2**	10	0.0009	9.03	42.34
**3**	1	N/A	N/A	31.12
**4**	11	0.11	7.34	55.57
**5**	5	4.00	9.34	32.41
**6**	4	4.6	10.18	50.97
**7**	12	0.21	8.98	41.64
**8**	5	0.039	14.11	34.98
**9**	12	0.08	14.56	52.57
**10**	6	0.48	16.09	44.47
**11**	5	0.42	10.18	42.29
**12**	4	6.83	12.67	29.14
**13**	3	9.26	9.32	40.93
**14**	14	0.18	13.17	34.95
**15**	5	0.80	8.90	57.82
**16**	5	1.07	9.50	32.72
**17**	10	0.10	12.8	34.47
**18**	7	0.16	14.3	38.47
**19**	4	0.70	19.57	31.21
**20**	8	0.41	9.70	35.00
**21**	4	5.50	13.8	39.01
**22**	3	7.36	13.4	29.01
**23**	9	0.29	9.70	31.60

The above table shows marker coverage used for the detection of QTL. With the exception of LG 3, which has one marker, the number of markers per LG ranges from 3-12 and the size of intervals between markers ranges from 0.1-19.57. The total length of each LG and the marker positions are based on the guppy linkage map previously published by Tripathi et al. (2009).

### Confirmation and fine-mapping of QTL on LG 14

**Figure 2:**
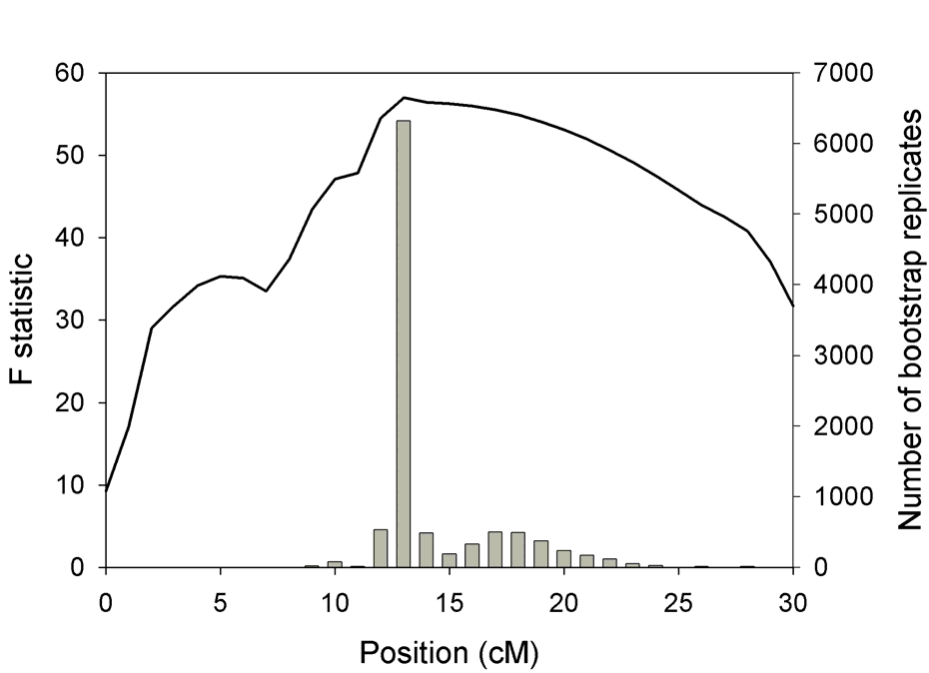
**Results of fine-mapping the susceptibility QTL on LG14**. Interval mapping using the genotypes from Cross 1 (BC) and Cross 2 (F2) has localized the QTL to approximately 13 cM (x-axis) on LG14. The solid line represents the results of interval mapping whereas the histogram represents the distribution of the estimated QTL peak from 10000 bootstrap replicates used to estimate the location of the QTL [22]; 95% of the bootstrap estimates fall within a region of 9 cM.

From Cross 2, 27 F1 were produced, none of which were curved. From the F1, four intercrosses were made. To localize the major QTL on LG14, we genotyped 175 F2 from Cross 2 with 12 guppy map markers between 0 and 24 cM [21], including 7 that were not informative in Cross 1 (Additional file 1). The parents of Cross 2 were homozygous for all markers. Interval analysis using genotype data from both crosses and the additional markers estimated the QTL location to be at 13 cM on LG14 (LOD 19.53), with a 95% confidence interval estimated by bootstrapping to be 9 cM [22] (**Figure **2). Calculation of the 95% confidence interval based on the two-LOD support interval yielded a slightly larger confidence interval, suggesting the region from 11 to 23 cM. Using the genotypes for Marker 0289 at 13 cM, this QTL explains approximately 82.6% of the phenotypic variation in Cross 2, and 54% of the difference between the parents.

**Figure 3:**
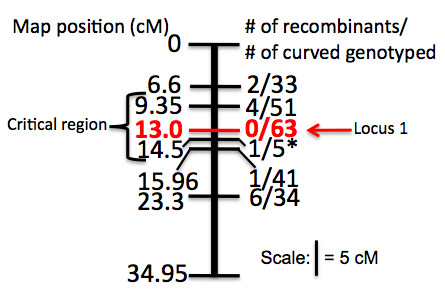
**Identification of susceptibility locus**. Curve predisposition is recessive. Therefore, curved individuals that were not homozygous for the allele from the curved parent (*i.e. *recombinants) at a given marker exclude that marker from the QTL region. By genotyping curved individuals at each marker within the QTL defined in Figure 2, we were able to identify a region in which all curved individuals are homozygous for the curved allele. Marker 0289 (at 13 cM) defines locus 1 and accounts for 100% of curve susceptibility. Flanking markers 0635 and 0381 at 9.35 and 14.5 cM are heterozygous in one or more curved individuals and so delimit the QTL region. *For the marker at 14.5 cM only the curved individuals who are heterozygous at 15.96 or 9.35 were genotyped.

No curved individuals were observed in the F1 populations of Cross 1 and Cross 2, suggesting that curve predisposition is recessive. Recessive inheritance was also confirmed by estimates of the additive and dominance effects from Cross 2, which were equal (1.08 in both cases). Therefore, curved individuals that were not homozygous for the allele from the curved parent at a given marker excluded that marker from the QTL region. In this way, we were able to narrow the QTL region further than by interval mapping (which does not assume that curved individuals are homozygous at the QTL). In Cross 2, Marker 0289 (at 13 cM) is homozygous for the curved allele in all curved individuals (and all non-curved individuals are either homozygous for the wild-type allele or are heterozygous), whereas flanking Markers 0635 and 0381(at 9.35 and 14.5 cM) are heterozygous in one or more curved individuals and so delimit the QTL region (**Figure **3). Thus, the QTL has been narrowed to a 5 cM region.

## Discussion

To identify genomic regions that are associated with idiopathic-type spinal curvature in the *curveback *guppy, we screened 84 backcross progeny with 367 guppy-specific markers to identify a major locus that accounts for curve susceptibility. In a second mapping cross between *curveback *and a more outbred lineage, the susceptibility locus was confirmed. The 57 non-curved F1 from mapping Crosses 1 and 2, estimates of allele effects from our mapping crosses, as well as evidence from the *curveback *pedigree (Gorman, *et al., *2007: F1 of the original pedigree cross are non-curved), confirm that the inheritance of curve susceptibility in *curveback *is recessive.

Localization of the susceptibility QTL on LG14 using additional F2 mapping cross progeny from the second cross identified a 5 cM interval containing a marker whose genotypes explain 100% of the susceptibility for curvature (*i.e., *comparing curved vs. non-curved individuals), 82.6% of the phenotypic variation in curve severity (*i.e, *qualitative score 0-4), and 54% of the difference between the parents. The estimated difference in curvature between curved homozygotes and heterozygotes at the LG14 QTL is slightly higher in Cross 2 (2.16) than in Cross 1 (1.39), but this difference could be due to different genetic backgrounds in the two crosses.

Based on synteny between the guppy and medaka genomes, we estimate that there are 100-150 genes in the QTL interval. Synteny between the guppy and medaka genomes has been established by Tripathi et al., (2009), using the same guppy markers as this QTL study (Additional file 1). Moreover, to confirm synteny for LG 14, we blasted all guppy LG14 EST and BAC end clone sequences (available on: http://www.ncbi.nlm.nih.gov/pubmed/) to the medaka genome using the BLAST algorithm (Ensembl release 57: http://www.ensembl.org). We found that the gene order for 17 out of 20 markers is conserved between guppy and medaka on LG 14, demonstrating chromosome-wide synteny between these species. Therefore, we searched the medaka region corresponding to the 5 cM Guppy QTL for candidate genes named in human studies of IS. We found that the melatonin 1B receptor gene (MTNR1B), which has been implicated as a candidate for curve predisposition in human IS, is contained in the QTL [23].

### The importance in identifying genes involved in spinal curvature

Spinal curvature is a prevalent and costly deformity among humans and teleosts. The estimated annual cost of treating children hospitalized with idiopathic scoliosis (IS) in the United States alone is over $3 billion. This cost estimate does not consider Scheuermann kyphosis, or adults with idiopathic-type spinal curvature who suffer from chronic back pain, contributing to the estimated $849 billion cost of treatment and lost wages associated with musculoskeletal disease [24]. Among teleosts, spinal column deformities reduce total production in the aquaculture industry substantially [12]. In contrast to humans, teleost curve phenotypes are less well characterized; although heritable curves are acknowledged to account for many cases among cultured stocks, whether these cases are from congenital defects or are idiopathic-type is often not known.

Despite the prevalence and impact of this type of deformity, the genetic architecture and specific genes involved are unknown. The current view is that human idiopathic-type scoliosis is a complex genetic disorder with multiple genes segregating in the population exhibiting complex genotype by environment interactions [25-30]. In aquaculture stocks, inheritance for spinal curvature has been described as Mendelian recessive or dominant, as well as polygenic, depending on how well the phenotype is characterized and what stock is considered (reviewed in [13], [16]). The guppy *curveback *phenotype has been extensively characterized so that the lineage can be applied as a model for understanding the biological context of heritable spinal curvature [1,7-9]. Future studies can use approaches to map QTL affecting shape based on digital photos [31,32], rather than the qualitative scale used in the present study. The identification of QTL in this study is a first step in understanding the genetics of this type of deformity and will lead to the identification of biological pathways associated with spinal integrity.

## Conclusions

We detected a major QTL associated with idiopathic-type spinal curvature in the *curveback *guppy, and identify this as a susceptibility locus. The locus acts in a recessive manner and accounts for a large portion of phenotypic variance. Fine mapping of the susceptibility locus has identified a 5 cM region that contains over 100 genes among which is the human candidate for IS, MTNR1B. The identification of genes for spinal curvature in *curveback *will determine the molecular pathway(s) leading to spinal curvature in the guppy, and these findings will provide insights into the etiopathogenesis of spinal curvature in humans and other teleost species.

## Methods

### Study population and phenotypic evaluation

The guppy, *P. reticulata, *is a live-bearing teleost native to streams in northeast South America that has been used as a genetic model since the 1920's. The *curveback *lineage was established from laboratory guppies that are derived from a wild population caught in Central Cumaná, Venezuela and raised under standardized conditions since 2000. The *curveback *lineage originated from a curved male crossed to a non-curved, unrelated female in 2003, followed by full-sib mating and backcrossing. Breeding pairs are maintained in 4L plastic aquaria, and offspring are separated into individual 600 ml plastic containers after birth. Laboratory fish are kept under standardized conditions as described in Gorman et al., (2007), in compliance with protocols approved by the Simon Fraser University Animal Care Facility and the Canadian Council on Animal Care (#763B-05).

The guppy spine is visible without magnification. Fish were scored from the side (for sagittal curvature) and above (for coronal curvature) while in a plastic view tank 4"long × 2"wide × 3"high. Since all affected individuals exhibit sagittal curvature, the degree of lordosis is used as a standard for comparison of curve magnitude. Evaluation of curve magnitude is based on scores defined by Gorman et al., (2007) in which 0 denotes no curvature and scores of 1 though 4 reflect increasing curve magnitudes. Fish were scored for curve magnitude after birth and then once a month until 3 months of age (sexual maturity is at approximately one month). To maintain consistency and precision in scores, the same individual evaluated all fish.

At 3 months past birth fish were culled and photographed on a light table with a digital camera (Toshiba PDR-3310, NYC, USA) under 3X magnification. As described in Gorman and Breden (2010), all fish were positioned on their side and photographs were taken from above so that the camera looked down on the sagittal profile of the fish. The qualitative adult score 0-4 was used for analysis [7]. Adult fish were frozen in buffered (EDTA) ethanol for genotyping. DNA was extracted from tissue using the DNeasy Blood and Tissue kit and protocol (Qiagen), and diluted as per specifications from Sequenom Inc. (see below).

### Crosses used for mapping QTL

To detect QTL associated with curvature, mapping Cross 1 was generated from an extremely curved male (Pmale1) from the *curveback *lineage mated with a non-curved and unrelated female from a lineage having no prior incidence of spinal curvature (Pfemale1). The maternal lineage is a mixture of laboratory fish derived from Central Cumaná, Venezuela and a pet store fancy guppy stock, and has been inbred (via sib mating) for 7 generations as part of another experiment. According to the qualitative scale for curve magnitude ([7]), Pmale1 had a magnitude of 4 (the highest curve magnitude), and Pfemale1 had a score of 0 (non-curved). From Cross 1 30 F1 offspring were produced, none of which were curved. Two non-curved F1 daughters were subsequently backcrossed to their curved father to obtain a total of 129 backcross (BC) mapping progeny. In addition, two non-curved F1 progeny were crossed to create 286 F2s that were used to estimate the distribution of curve magnitude, but were not used for genome-wide mapping.

For confirmation of the QTL detected in Cross1 and for fine mapping, additional offspring were generated from a second mapping cross. Cross 2 is an extremely curved *curveback *female (Pfemale2) crossed to a non-curved male from the QUL lineage (Pmale2). The QUL lineage is derived from the lower Quare River in Trinidad and was chosen as the outcross lineage because it exhibits a high rate of polymorphism in molecular markers compared to the Central Cumaná strain [33] from which the *curveback *lineage was derived. The Pfemale2 is derived from the same *curveback *lineage as Pmale1, but is the result of further generations of inbreeding. From Cross 2, 27 non-curved F1offspring were produced, from which we made four crosses to generate a total of 259 F2 mapping progeny.

### Marker selection and QTL mapping

The guppy genome consists of 23 haploid chromosomes [34], with a genetic map distance estimated to be 899 cM, with no linkage group (LG) longer than 58 cM [21]. The 2 parentals, 9 F1 males and females (including those used in production of BC and F2 offspring), and 53 non-curved and 31 curved BC offspring, were genotyped by Sequenom, Inc., using 189 EST-based (expressed sequence tag) and 178 genomic guppy-specific SNP (single nucleotide polymorphism) markers [21]. These SNP markers are optimized for high-throughput genotyping by Sequenom Inc. using mass spectrometry. Markers were chosen based on their map positions published in Tripathi *et al., *(2009), with the goal of creating a map with 10 cM resolution.

To detect QTL that are associated with curvature in Cross 1, and to confirm the location of the major QTL in Cross 2, we performed interval mapping using GridQTL at http://www.gridqtl.org.uk/[35,36]. Grid QTL evaluates the statistical support for the presence of a QTL at positions throughout the genome using least squares regression, which has been shown to be robust for use with non-normal and discrete characters, including binary traits [37,38]. This program is able to analyze outbred populations; although the parental lines used in this study have been inbred for several generations, they are not homozygous at all marker loci. Because the Cross 1 parents were heterozygous at numerous markers, and because of the smaller sample sizes for the mapping crosses, we used genetic distances from Tripathi et al. (2009).

We first conducted analyses under the single locus model, and then under the two QTL model. For putative QTL, the substitution effect (difference between curved parent allele homozygotes and heterozygotes) was estimated in Cross 1 and additive and dominance effects were estimated in Cross 2. Sex was included as a fixed effect, and we also tested for an interaction between putative QTL and sex. To determine significance thresholds, one thousand permutations per chromosome (linkage group) were carried out for each trait to determine the distribution of the *F*-statistic under the null hypothesis that no QTL was segregating on that chromosome [39]. For markers that could not be assigned to a linkage group for interval analysis, association with curvature was tested using individual marker genotypes and a one-way ANOVA. We used a significance threshold of 0.05 for all ANOVAs, which were conducted using JMP statistical software for MacOSX, Version 7.0, SAS Institute, INC., Cary, NC, USA.

### Fine-mapping of a major effect QTL

The initial genome screen of Cross 1 detected a major effect QTL on LG14 (see Results) and so we genotyped 9 additional SNP markers in this region [21] by either direct sequencing of PCR products or PCR based RFLP [36] (Additional file 1 for marker numbers). However, further localization of the QTL was limited due to a lack of markers polymorphic between the parental strains in Cross 1. Using the F2s from Cross 2, we genotyped 175 individuals at markers on LG 14, including 7 markers that were not polymorphic in Cross 1 [21] (see supplemental data), by either direct sequencing of PCR products or PCR- based RFLP [40].

## Authors' contributions

KFG and FB participated in the project conception and oversight. KFG was also in charge of mapping crosses and phenotyping. JP and RA were in charge of DNA extraction, and JP performed all PCR and RFLP procedures and sequence analysis for Cross 2. CD and DW provided the guppy specific markers. KFG, FB, and JKC performed statistical analyses. KFG and JKC drafted the manuscript and FB contributed to revisions for the final draft. All authors have read and approved the final manuscript.

## Supplementary Material

Additional file 1 **Polymorphic markers used for mapping**. The supplemental file provides the markers that were polymorphic in *curveback *mapping crosses. All marker numbers and genetic locations are from the guppy linkage map (Tripathi, *et al., *2009). Markers in bold were genotyped by either direct sequencing or by RFLP of PCR products. File is in Excel 97-2004 format. Click here for file
